# Target Capturing Control for Space Robots with Unknown Mass Properties: A Self-Tuning Method Based on Gyros and Cameras

**DOI:** 10.3390/s16091383

**Published:** 2016-08-30

**Authors:** Zhenyu Li, Bin Wang, Hong Liu

**Affiliations:** State Key Laboratory of Robotics and System, Harbin Institute of Technology, Harbin 150000, China; dlrhitlab@aliyun.com

**Keywords:** free-floating space robot, autonomous target capturing, space robot modeling, self-tuning control, mass property estimation

## Abstract

Satellite capturing with free-floating space robots is still a challenging task due to the non-fixed base and unknown mass property issues. In this paper gyro and eye-in-hand camera data are adopted as an alternative choice for solving this problem. For this improved system, a new modeling approach that reduces the complexity of system control and identification is proposed. With the newly developed model, the space robot is equivalent to a ground-fixed manipulator system. Accordingly, a self-tuning control scheme is applied to handle such a control problem including unknown parameters. To determine the controller parameters, an estimator is designed based on the least-squares technique for identifying the unknown mass properties in real time. The proposed method is tested with a credible 3-dimensional ground verification experimental system, and the experimental results confirm the effectiveness of the proposed control scheme.

## 1. Introduction

With development of space technology, modern space missions are becoming much more complex than ever before, including tasks such as on-orbit refueling, damaged module replacement, target satellite capture, etc. To complete such challenging missions, many attempts have been made to design special robots for space applications. The free-floating space robot was invented as a particular kind of robotic system for on-orbit satellite recovery and maintenance. It employs a free-flying satellite and robotic manipulators mounted on it, so that it can perform on-orbit approaching and target operating tasks. For extending the service life, the position and attitude of the carrier satellite are totally uncontrolled during operations. There have been several successful free-flying space robots, such as Engineering Test Satellite VII (ETS–VII) which is the first space robot project that successfully captured an autonomous on-orbit target [1,2,3] and the orbital express plan of NASA [4,5,6], etc. Other free-floating space robot projects have also been carried out as described in [7,8].

The kinematics, dynamics and control of a free-floating space robot are much more complex compared to their ground counterparts, because of the free-floating base satellite. Since the base satellite is free-floating, any robot arm movements may result in changes to the base satellite position and attitude due to the dynamical interactions between the manipulator and the satellite carrier [9,10]. To solve this problem, a modeling technique was proposed by Umetani and Yoshida [11]. They introduced the momentum conservation law into the space robot kinematics formulation, and put forward the concept of the generalized Jacobian matrix. Another successful modeling technique known as the Virtual Manipulator (VM) technique was proposed by Vafa and Dubowsky [12]. They simplified the kinematics equation by decoupling the system centroid translational degrees of freedom. The VM technique sets up a virtual ground-fixed manipulator to describe the motions of the free-floating space robot. Dubowsky and Papadopoulos compared the structure of the motion equations for space robots and ground-fixed robots in [13]. They concluded that if the carrier satellite attitude can be measured or calculated, almost all the ground robot control strategies can be applied to a free-floating space robot. Based on these models and concepts, a number of control methods have been proposed for free-floating space robots. A variable structure control strategy was presented by Fang [14], where a neural network controller is used as the dynamic compensator. Huang et al. studied the tethered space robot (TSR), and presented several trajectory planning and control methods [15,16,17]. To stabilize the TSR during capture impact with target, Huang derived the impact dynamic model for target capturing and an adaptive robust controller was designed accordingly [18]. Because the collision during the capture and the original target rotation leads to a tumbling of the tethered space robot–target combination system, a robust adaptive backstepping controller was designed to realize stabilization after the target is captured [19]. Huang also investigated the spacecraft attitude takeover control for extending the life time of fuel-exhausted spacecraft [20]. He designed a reconfigurable control system handling the attitude control problem including the mass property changes. Pathak proposed a robust overwhelming control method for space robot and verified it by numerical simulations [21]. Tsuchiya studied the satellite attitude dynamics of space robot and proposed an attitude control scheme based on the reaction wheels [22]. Rastegari and Moosavian suggested a multiple impedance control approach for free-flying space robots to track the path and tune the inner forces at the same time [23]. Zarafshan and Moosavian investigated the dynamics of space robot with flexible elements and proposed a hybrid suppression control method [24]. Considering that pure motion control is not applicable for the satellite-capturing tasks, sensor-based control methods are also applied in space robot engineering. One of the most effective sensor-based robot control strategies is the visual servoing. Instead of the “looking” then “moving” mode which combines the visual sensor and the robotic system in an open-loop fashion, visual servoing introduces a visual feedback control loop to increase the accuracy of the overall system. The basic conceptual framework of visual servoing for robotic manipulators is introduced in Hutchinson’s article [25]. In this paper, two major classes of visual servoing systems were discussed in detail. Chaumette and Hutchinson described the basic approaches and advanced techniques of visual servoing in [26,27] and the performance and stability issues of the two visual servoing schemes were discussed. The visual servoing techniques have also been approved in space robot engineering by several scholars as reported in [28,29,30,31].

Although a lot of achievements have been made in space robot modelling and control, there are still several issues to be considered in practical engineering. One practical problem is that the mass properties of the carrier satellite keep changing due to the fuel consumption, solar panel adjustments, carrying a captured payload with undetermined mass, etc. These unknown properties are critical and recognized as a challenging problem in space robot target capture control. Since few efforts have been made for controller designing of space robots with unknown satellite mass properties, the undetermined properties of robotic systems are need to be known in advance. Yoshida and Abiko presented an approach for determining space robot inertia parameters [32]. Their identification algorithm is designed based on the conservation of momentum and the gravity gradient torque. In this method, only the reaction wheel motion rates need to be measured. Ma and Dang proposed another method to identify the carrier satellite inertia properties [33]. By using the linear and angular satellite velocities, this issue is treated as a linear identification problem. According to the existing literatures, the unknown satellite mass properties are generally determined based on the conservation-of-momentum principle. The largest advantages of such identification methods are: (1) they don’t consume any fuel because the properties are estimated by manipulator motions; (2) since the accelerations and forces aren’t directly involved in the calculation, in theory, only velocities need to be measured, which are generally less noisy. However, several difficulties still must be considered in identifying the satellite mass, which plays a very important role in space robot dynamics. One difficulty is that the carrier-satellite mass has quite low sensitivity to the angular motions of the satellite, which means it can’t be identified only by reaction wheel motion rates or gyro signals. The other problem is that although the satellite mass can be identified by the linear velocities of the carrier satellite and no acceleration or force is directly involved in the computation, in fact, the linear velocities can’t be measured as easily as the angular velocities. In engineering, linear velocities are usually integrated from accelerometer data, which brings drifting errors. Based on such considerations, new identification methods should be proposed trying to handle these issues.

In this paper, a new identification strategy is proposed to estimate the unknown mass properties without computing the linear velocity of the carrier satellite. The eye-in-hand camera signals, which are commonly used in space robot target capture, are adopted as well as the gyro data for identifying both the satellite mass and the centroid position. Because the unknown mass properties are estimated in real time, the self-tuning control scheme is applied to handle this capturing control problem including unknown parameters. The whole approach is established based on a new space robot model for reducing the computation complexity. The rest of this paper is organized as follows: Section 2 describes the new space robot modeling method proposed in this paper; Section 3 designs the self-tuning scheme and presents the new identification method; Section 4 introduces the ground verification experimental system and gives the simulation results; and the results are discussed in Section 5.

## 2. Free-Floating Space Robot Kinematics Modeling

**Figure 1:**
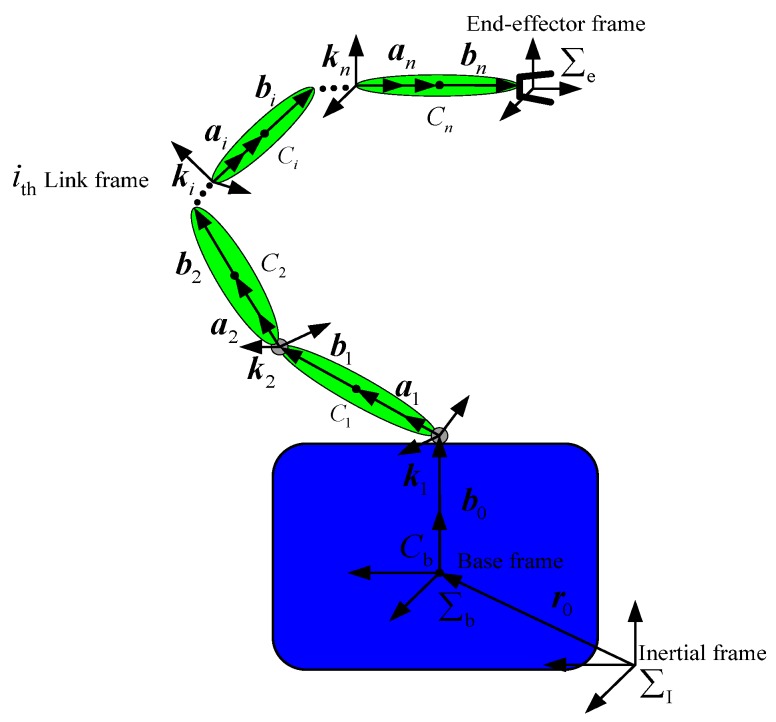
Space robot system.

The space robot system, consisting of a carrier satellite and a space manipulator mounted on it, is shown in Figure 1.

$a_{i}$ is the position vector from the $i_{\text{th}}$ joint of the robotic arm to the centroid of the $i_{\text{th}}$ link. $b_{i}$ is the position vector from the centroid of the $i_{\text{th}}$ link to the $i+1_{\text{th}}$ joint. $k_{i}$ is the rotation vector of the $i_{\text{th}}$ joint. $r_{0}$ represents the position of the carrier satellite. $b_{0}$ is the position vector from the carrier satellite centroid to the first joint. 

According to Figure 1, the position of space robot end-effector can be determined as:

(1) $$P_{e}=r_{0}+\sum_{i=1}^{n}a_{i}+\sum_{i=0}^{n}b_{i}$$

where $r_{0}$ is not a constant vector, because the carrier satellite is free-floating in space. The centroid position of the space robot system can be expressed as:

(2) $$r_{g}=\frac{\sum_{i=1}^{n}m_{i}r_{i}+m_{0}r_{0}}{M}$$

where $m_{i}$ is the mass of the $i_{\text{th}}$ link; $m_{0}$ is the mass of the carrier satellite; $r_{i}$ is the centroid position of the $i_{\text{th}}$ link; M is the total mass of the space robot system. They can be obtained by:

(3) $$r_{i}=r_{0}+\sum_{k=0}^{i-1}b_{k}+\sum_{k=1}^{i}a_{k}$$

(4) $$M=\sum_{i=0}^{n}m_{i}$$

Assuming that there is no external force acting on the space robot, the centroid position of the space robot system will not change. By Equations (2)–(4), the position of the carrier satellite can be determined as:

(5) $$r_{0}=r_{g}-\frac{1}{M}\left(\sum_{i=1}^{n}\left(\sum_{k=i}^{n}m_{k}\right)a_{i}\right)-\frac{1}{M}\left(\sum_{i=0}^{n-1}\left(\sum_{k=i+1}^{n}m_{k}\right)b_{i}\right)$$

According to Equation (5), a hypothetical manipulator can be set up to describe the linear motion of the carrier satellite. The length vector from the $i_{\text{th}}$ joint of the hypothetical manipulator to the joint i+1, is defined as:

(6) $$l_{i^{\prime }}=\{\begin{array}{ll} \gamma {\overset{⌢}{b}}_{i} & i=0\\ \gamma \left({\overset{⌢}{a}}_{i}+{\overset{⌢}{b}}_{i}\right) & 0<i<n\\ \gamma {\overset{⌢}{a}}_{i} & i=n \end{array}$$

where:

(7) $$\left\{ \begin{array}{l} {\overset{⌢}{a}}_{i}=\left(\sum_{k=i}^{n}m_{k}\right)a_{i}\\ {\overset{⌢}{b}}_{i}=\left(\sum_{k=i+1}^{n}m_{k}\right)b_{i}\\ \gamma =-\frac{1}{M} \end{array}\right.$$

Attaching the hypothetical manipulator to the centroid of the space robot system, the end position of the hypothetical manipulator can be calculated as:

(8) $$\begin{array}{ll} P_{e^{\prime }} & =r_{g}+\gamma \sum_{i=1}^{n}{\overset{⌢}{a}}_{i}+\gamma \sum_{i=0}^{n-1}{\overset{⌢}{b}}_{i}\\ & =r_{0} \end{array}$$

**Figure 2:**
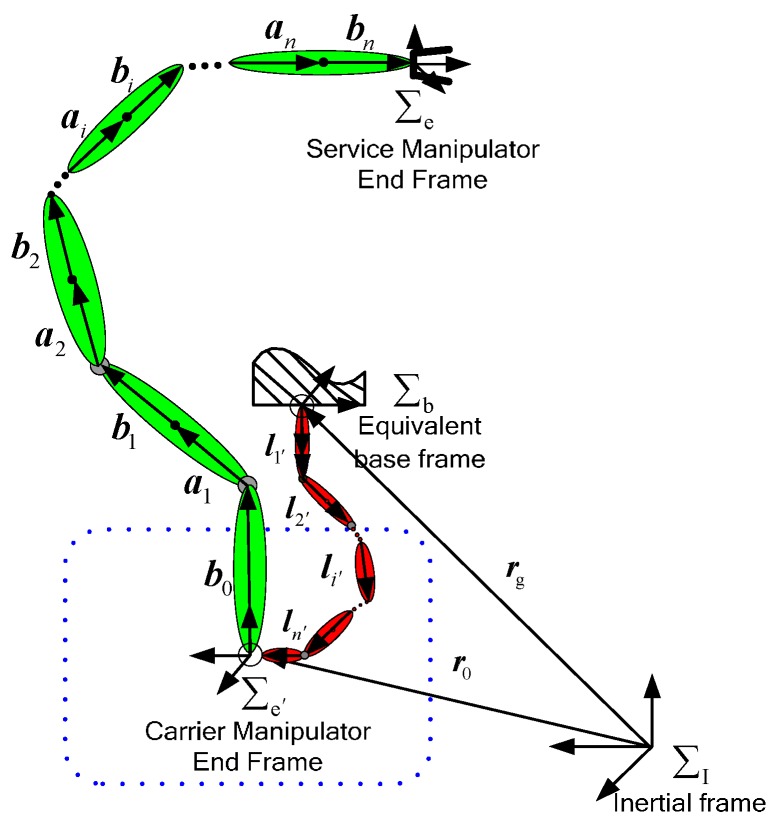
Equivalent robotic system.

According to Equation (8), the carrier satellite position can be obtained by the hypothetical manipulator motion. In this case, the hypothetical manipulator is referred as the carrier manipulator. The position of the space robot end-effector can be expressed as:

(9) $$\begin{array}{ll} P_{e} & =r_{0}+\sum_{i=1}^{n}a_{i}+\sum_{i=0}^{n}b_{i}\\ & =P_{e^{\prime }}+L_{0e} \end{array}$$

where $L_{0e}$ is the position vector from the carrier satellite centroid to the space robot end. If the carrier satellite is considered as the 0_th_ link, the space robot can be considered as another hypothetical manipulator mounted on the carrier manipulator end. Since it performs the same operation motions as the space manipulator, it is referred as the service manipulator. Consequently, the free-floating space robot system is equivalent to a hypothetical ground-fixed robotic system including two manipulators. The hypothetical robotic system can be described as Figure 2.

According to Equation (9), the velocity vector of the service manipulator end, representing the space robot end-effector, can be expressed as:

(10) $$v_{e}={\dot{P}}_{e}={\dot{P}}_{e^{\prime }}+{\dot{L}}_{0e}$$

where:

(11) $${\dot{L}}_{0e}=\sum_{i=1}^{n}{\dot{a}}_{i}+\sum_{i=0}^{n}{\dot{b}}_{i}$$

(12) $${\dot{P}}_{e^{\prime }}=\gamma \sum_{k=1}^{n}{\dot{\overset{⌢}{a}}}_{k}+\gamma \sum_{k=0}^{n-1}{\dot{\overset{⌢}{b}}}_{k}$$

and ${\dot{a}}_{i}$, as well as ${\dot{b}}_{i}$ can be determined by:

(13) $$\{\begin{array}{l} {\dot{a}}_{i}=\omega_{i}\times a_{i}\\ {\dot{b}}_{i}=\omega_{i}\times b_{i} \end{array}$$

where $\omega_{i}$ is the angular velocity of the *i*_th_ link and can be described as:

(14) $$\omega_{i}=\omega_{0}+\sum_{k=1}^{i}k_{k}{\dot{\theta }}_{k}$$

and $\omega_{0}$ is the satellite angular velocity; ${\dot{\theta }}_{k}$ is the angle velocity of the $k_{\text{th}}$ link. Substituting Equations (13) and (14) into Equation (11) gives:

(15) $$\begin{array}{ll} {\dot{L}}_{0e} & =\sum_{i=1}^{n}\left[\left(\omega_{0}+\sum_{k=1}^{i}k_{k}{\dot{\theta }}_{k}\right)\times a_{i}\right]+\sum_{i=1}^{n}\left[\left(\omega_{0}+\sum_{k=1}^{i}k_{k}{\dot{\theta }}_{k}\right)\times b_{i}\right]+\omega_{0}\times b_{0}\\ & =\omega_{0}\times \left(\sum_{i=0}^{n}b_{i}+\sum_{i=1}^{n}a_{i}\right)+\sum_{i=1}^{n}\left[k_{i}\times \left(\sum_{k=i}^{n}b_{k}+\sum_{k=i}^{n}a_{k}\right){\dot{\theta }}_{i}\right] \end{array}$$

Defining $L_{ie}$ as the position vector from the $i_{\text{th}}$ joint to the service manipulator end, it can be determined as:

(16) $$L_{ie}=\begin{array}{cc} \sum_{k=i}^{n}a_{k}+\sum_{k=i}^{n}b_{k} & n\geq i>1 \end{array}$$

According to Equations (15) and (16), ${\dot{L}}_{0e}$ is derived as:

(17) $$\begin{array}{ll} {\dot{L}}_{0e} & =\omega_{0}\times L_{0e}+\sum_{i=1}^{n}\left[k_{i}\times L_{ie}{\dot{\theta }}_{i}\right]\\ & =\left(J_{r}-{\tilde{b}}_{0}\right)\omega_{0}+J_{m}\dot{\theta } \end{array}$$

where:

$$\{\begin{array}{l} J_{r}=-{\tilde{L}}_{1e}\\ J_{m}=\left[\begin{array}{lllll} k_{1}\times L_{1e} & \cdots & k_{i}\times L_{ie} & \cdots & k_{n}\times L_{ne} \end{array}\right] \end{array}$$

According to Equations (7) and (13), ${\dot{\overset{⌢}{a}}}_{k}$ and ${\dot{\overset{⌢}{b}}}_{k}$ can be defined by:

(18) $$\{\begin{array}{l} {\dot{\overset{⌢}{a}}}_{k}=\omega_{k}\times \left(\sum_{q=k}^{n}m_{q}\right)a_{k}=\omega_{k}\times {\overset{⌢}{a}}_{k}\\ {\dot{\overset{⌢}{b}}}_{k}=\omega_{k}\times \left(\sum_{q=k+1}^{n}m_{q}\right)b_{k}=\omega_{k}\times {\overset{⌢}{b}}_{k} \end{array}$$

Substituting Equations (14) and (18) into Equation (12), we have:

(19) $$\begin{array}{ll} {\dot{P}}_{e^{\prime }} & =\gamma \sum_{k=1}^{n}\left[\left(\omega_{0}+\sum_{i=1}^{k}k_{i}{\dot{\theta }}_{i}\right)\times {\overset{⌢}{a}}_{k}\right]+\gamma \sum_{k=1}^{n-1}\left[\left(\omega_{0}+\sum_{i=1}^{k}k_{i}{\dot{\theta }}_{i}\right)\times {\overset{⌢}{b}}_{k}\right]+\gamma \omega_{0}\times {\overset{⌢}{b}}_{0}\\ & =\gamma \omega_{0}\times \left(\sum_{k=0}^{n-1}{\overset{⌢}{b}}_{k}+\sum_{k=1}^{n}{\overset{⌢}{a}}_{k}\right)+\gamma \sum_{i=1}^{n}\left[k_{i}\times \left(\sum_{k=i}^{n-1}{\overset{⌢}{b}}_{k}+\sum_{k=i}^{n}{\overset{⌢}{a}}_{k}\right){\dot{\theta }}_{i}\right] \end{array}$$

Defining $L_{i^{\prime }e^{\prime }}$ as the position vector from the $i_{\text{th}}$ joint to the end of the carrier manipulator, it can be expressed as:

(20) $$L_{i^{\prime }e^{\prime }}=\gamma {\overset{⌢}{L}}_{ie}$$

where:

(21) $${\overset{⌢}{L}}_{ie}=\{\begin{array}{ll} {\overset{⌢}{a}}_{n} & i=n\\ \left(\sum_{k=i}^{n}{\overset{⌢}{a}}_{k}+\sum_{k=i}^{n-1}{\overset{⌢}{b}}_{k}\right) & 0<i<n\\ \left(\sum_{k=1}^{n}{\overset{⌢}{a}}_{k}+\sum_{k=0}^{n-1}{\overset{⌢}{b}}_{k}\right) & i=0 \end{array}$$

According to Equations (19) and (20), ${\dot{P}}_{e^{\prime }}$ can be further derived as:

(22) $$\begin{array}{l} {\dot{P}}_{e^{\prime }}=\omega_{0}\times L_{0^{\prime }e^{\prime }}+\sum_{i=1}^{n}\left[k_{i}\times L_{i^{\prime }e^{\prime }}{\dot{\theta }}_{i}\right]\\ =\left(J_{r^{\prime }}-\gamma M_{m}{\tilde{b}}_{0}\right)\omega_{0}+J_{m^{\prime }}\dot{\theta } \end{array}$$

where:

$$\{\begin{array}{l} J_{r^{\prime }}=-{\tilde{L}}_{1^{\prime }e^{\prime }}\\ J_{m^{\prime }}=\left[\begin{array}{lllll} k_{1}\times L_{1^{\prime }e^{\prime }} & \cdots & k_{i}\times L_{i^{\prime }e^{\prime }} & \cdots & k_{n}\times L_{n^{\prime }e^{\prime }} \end{array}\right] \end{array}$$

and $M_{m}$ is the total mass of the manipulator, known as:

(23) $$M_{m}=\sum_{i=1}^{n}m_{i}$$

Defining the Jacobian matrices ${\overset{⌢}{J}}_{r}$ and ${\overset{⌢}{J}}_{m}$ as:

$$\{\begin{array}{l} {\overset{⌢}{J}}_{r}=-{\tilde{\overset{⌢}{L}}}_{1e}=\frac{1}{\gamma }J_{r^{\prime }}\\ {\overset{⌢}{J}}_{m}=\left[\begin{array}{lllll} k_{1}\times {\overset{⌢}{L}}_{1e} & \cdots & k_{i}\times {\overset{⌢}{L}}_{ie} & \cdots & k_{n}\times {\overset{⌢}{L}}_{ne} \end{array}\right]=\frac{1}{\gamma }J_{m^{\prime }} \end{array}$$

as a result, ${\dot{P}}_{e^{\prime }}$ also takes the following form:

(24) $${\dot{P}}_{e^{\prime }}=\gamma \left[\left({\overset{⌢}{J}}_{r}-M_{m}{\tilde{b}}_{0}\right)\omega_{0}+{\overset{⌢}{J}}_{m}\dot{\theta }\right]$$

In Equation (24), the unknown satellite mass is only contained in the parameter γ. According to Equations (17) and (24), the differential kinematics equation of free-floating space robot translational motion can be expressed as:

(25) $$v_{e}=\left[J_{r}+\gamma {\overset{⌢}{J}}_{r}-\left(1+\gamma M_{m}\right){\tilde{b}}_{0}\right]\omega_{0}+\left(J_{m}+\gamma {\overset{⌢}{J}}_{m}\right)\dot{\theta }$$

where $J_{m}$ and ${\overset{⌢}{J}}_{m}$ are exactly the same as the Jacobian matrix of ground-fixed manipulators.

According to Equation (25), two linear velocities are defined as:

(26) $$\{\begin{array}{l} v_{\text{eo}}=J_{m}\dot{\theta }\\ v_{\text{ec}}={\overset{⌢}{J}}_{m}\dot{\theta } \end{array}$$

where $v_{\text{eo}}$, as well as $v_{\text{ec}}$ can be considered as the linear velocity of a ground-fixed manipulator. Consequently, the end-effector velocity of space robot can be further expressed as:

(27) $$v_{e}=v_{\text{eo}}+\omega_{0}\times L_{1e}+\gamma \left(v_{\text{ec}}+\omega_{0}\times {\overset{⌢}{L}}_{1e}\right)+\omega_{0}\times b_{0}+\gamma M_{m}\omega_{0}\times b_{0}$$

The main advantage in using Equation (27) is that the unknown satellite mass properties will not be involved in the calculation of $v_{\text{eo}}$ and $v_{\text{ec}}$, and only two parameters, γ and $b_{0}$ relating to the undetermined satellite mass and centroid are to be determined. Accordingly, in this paper, γ will be estimated instead of identifying the satellite mass. $m_{0}$ can still be determined by Equations (4) and (7) if necessary.

The proposed modeling method is derived by describing the carrier satellite translational motions with a hypothetical manipulator fixed on the centroid of the robotic system and taking the free-floating space robot as an equivalent ground-fixed manipulator system. Comparing with existing modeling methods presented in [11] and [12], several advantages of using this new method are described as follows:

(1) Since the free-floating space robot system is kinematically equivalent to the hypothetical robotic system, this new space robot model is not complex to compute because only ground-fixed robot kinematics are involved in the calculations.
(2) In the new translational-motion equations, the undetermined carrier satellite mass, which is a challenge in parameter estimating as suggested in [32], only impacts a constant factor, namely *γ*. Accordingly, this new modeling method is more convenient in identifying the satellite mass properties.

## 3. Self-Tuning Control Designing

Because the mass properties of the carrier satellite are changing throughout the whole service life, precise target capturing of free-floating space robots is considered as a challenging problem. For improving the control performance, a self-tuning target capturing control scheme is applied by adopting the eye-in-hand camera and gyros. The self-tuning control concept is obtained based on the certainty equivalence principle. By coupling a motion controller with an online parameter estimator, the self-tuning controller can perform simultaneous identification of unknown properties. Accordingly, instead of the unknown true values, the controller parameters are determined by the estimations.

**Figure 3:**
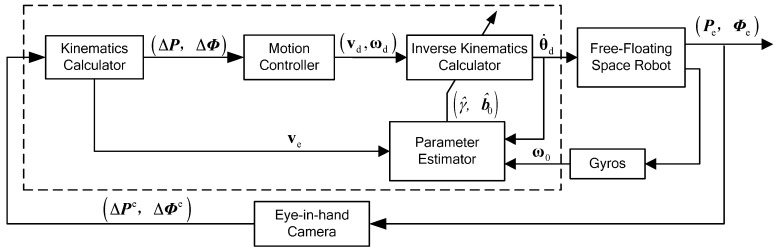
Block diagram of space robot self-tuning control scheme.

Because the dynamic and kinematic parameters of the space manipulator mounted on the carrier satellite are all constants, the satellite mass properties can be estimated in real time by end-effector translations and satellite rotations. As a consequence, the proposed space robot self-tuning control scheme is as shown in Figure 3.

The self-tuning control operation is described as follows: the motion controller plans the space robot end-effector motion based on the current relative position and attitude after transforming the camera signals to the inertial frame. At each time instant, a set of property estimations identified by the parameter estimator are sent to the inverse kinematics calculator, which is obtained from the past joint motions and the sensor data from eye-in-hand camera and gyros. Based on these estimated parameters and desired end-effector motions, the joint trajectories are planned by the space robot inverse kinematics calculator. The free-floating space robot joints move following the control input and generate a new output, updating the input data of the parameter estimator and motion controller.

### 3.1. Kinematics Calculator Designing

Because the outputs of the eye-in-hand camera are the relative position and attitude at the end-effector frame, a kinematics calculator is applied to transform the camera signals to the inertial frame. The relative position and attitude at the inertial frame are computed as:

(28) $$\{\begin{array}{l} \Delta P=A_{e}\left(\theta ,\psi \right)\Delta P^{e}\\ \Delta \Phi =A_{e}\left(\theta ,\psi \right)\Delta \Phi^{e} \end{array}$$

where $A_{e}$ is the rotation matrix; $\Delta P^{e}$ and $\Delta \Phi^{e}$ are the relative position and attitude at the end-effector frame; ψ is the satellite attitude. Based on the Euler axis/angle, ΔΦ is expressed as:

(29) ΔΦ=χρ

where χ is the Euler rotation angle; ρ is the Euler equivalent axis.

The linear end-effector velocity, which is applied as the parameter estimator input, is also computed by this calculator as:

(30) $$v_{e}=-\Delta \dot{P}+v_{t}$$

where $v_{t}$ is the linear velocity of the target satellite, which is assuming to be known or identified by other approaches.

### 3.2. Motion Controller Designing

Defining the relative pose as the control error of the free-floating space robot, it is written as:

(31) $$e=\left[\begin{array}{c} \Delta P\\ \Delta \Phi \end{array}\right]$$

The ideal feed-back response of e is designed as follows:

(32) $$Ke+\dot{e}=0$$

where K is the matrix of control factors reflecting performance specifications. According to Equations (31) and (32), the motion controller of the free-floating space robot is given by ignoring the target rotation as:

(33) $$\left[\begin{array}{c} v_{d}\\ \omega_{d} \end{array}\right]=Κ\left[\begin{array}{c} \Delta P\\ \Delta \Phi \end{array}\right]+\left[\begin{array}{c} v_{t}\\ 0 \end{array}\right]$$

### 3.3. Inverse Kinematics Calculating

According to Equation (25), the carrier satellite angular velocity is to be determined for performing inverse kinematics calculations. There are several ways to obtain the satellite angular velocity. One way is to substitute the angular momentum conservation equation into the kinematics equation. This, however, requires cumbersome computations. Another way is measuring the angular motion directly by gyros, which is especially convenient because almost all satellites are equipped with gyroscopes. Another advantage in adopting gyro information is that it will be unnecessary to identify the satellite inertia tensor matrix for calculating the inverse kinematics. Defining the measured satellite angular velocity as ${\bar{\omega }}_{0}$, the angular velocity of space robot end-effector is calculated as:

(34) $$\omega_{e}={\bar{\omega }}_{0}+J_{\omega }\dot{\theta }$$

where:

(35) $$J_{\omega }=\left[\begin{array}{ccccc} k_{1} & \cdots & k_{i} & \cdots & k_{1} \end{array}\right]$$

According to Equations (25) and (34), we have the following differential kinematics equation of the free-floating space robot as:

(36) $$\left[\begin{array}{c} v_{e}\\ \omega_{e} \end{array}\right]=\left[\begin{array}{c} J_{r}+\gamma {\overset{⌢}{J}}_{r}-\left(1+\gamma M_{m}\right){\tilde{b}}_{0}\\ E \end{array}\right]{\bar{\omega }}_{0}+\left[\begin{array}{c} J_{m}+\gamma {\overset{⌢}{J}}_{m}\\ J_{\omega } \end{array}\right]\dot{\theta }$$

Consequently, the desired manipulator joint velocities can be obtained as:

(37) $${\dot{\theta }}_{d}={\left[\begin{array}{c} J_{m}+\gamma {\overset{⌢}{J}}_{m}\\ J_{\omega } \end{array}\right]}^{-1}\left(\left[\begin{array}{c} v_{d}\\ \omega_{d} \end{array}\right]-\left[\begin{array}{c} J_{r}+\gamma {\overset{⌢}{J}}_{r}-\left(1+\gamma M_{m}\right){\tilde{b}}_{0}\\ E \end{array}\right]{\bar{\omega }}_{0}\right)$$

By Equation (37), once the mass properties, namely γ and $b_{0}$, are determined, space manipulator joint motions can be planned.

### 3.4. Mass Property Estimating

In this section, a real time estimator is proposed identifying the unknown mass properties relating to the inverse kinematics calculation. According to Equation (37), only γ and $b_{0}$ are to be estimated which indicate the system total mass and the satellite centroid position, respectively. Defining the linear velocity of the end-effector as a function of γ and $b_{0}$, it gives:

(38) $$v_{e}=f\left(\begin{array}{cc} \gamma , & b_{0} \end{array}\right)$$

Note that the unknown parameters γ and $b_{0}$ appear nonlinearly in Equation (38), linear identification techniques cannot be applied directly.

The estimation error is defined as:

(39) $$e={\hat{v}}_{e}-v_{e}$$

where $v_{e}$ is the true value of space robot end-effector velocity obtained by the eye-in-hand camera; ${\hat{v}}_{e}$ is the calculation employing the estimated mass properties $\hat{\gamma }$ and ${\hat{b}}_{0}$. Defining temporarily identified mass properties as ${\hat{\gamma }}_{\text{tem}}$ and ${\hat{b}}_{0\text{tem}}$, according to Equation (38), e can be linearized as:

(40) $$e=\left[f\left(\begin{array}{cc} {\hat{\gamma }}_{\text{tem}}, & {\hat{b}}_{0\text{tem}} \end{array}\right)+\frac{\partial f\left(\begin{array}{cc} {\hat{\gamma }}_{\text{tem}}, & {\hat{b}}_{0\text{tem}} \end{array}\right)}{\partial \gamma }\Delta \hat{\gamma }+\frac{\partial f\left(\begin{array}{cc} {\hat{\gamma }}_{\text{tem}}, & {\hat{b}}_{0\text{tem}} \end{array}\right)}{\partial b_{0}}\Delta {\hat{b}}_{0}\right]-v_{e}$$

where:

(41) $$\{\begin{array}{l} \Delta \hat{\gamma }=\hat{\gamma }-{\hat{\gamma }}_{\text{tem}}\\ \Delta {\hat{b}}_{0}={\hat{b}}_{0}-{\hat{b}}_{0\text{tem}} \end{array}$$

According to Equation (27), $\frac{\partial f\left(\begin{array}{cc} {\hat{\gamma }}_{\text{tem}}, & {\hat{b}}_{0\text{tem}} \end{array}\right)}{\partial \gamma }$ can be simply determined as:

(42) $$\frac{\partial f\left(\begin{array}{cc} {\hat{\gamma }}_{\text{tem}}, & {\hat{b}}_{0\text{tem}} \end{array}\right)}{\partial \gamma }=v_{\text{ec}}+\omega_{0}\times {\overset{⌢}{L}}_{1e}+M_{m}\omega_{0}\times {\hat{b}}_{0\text{tem}}$$

Defining a new robot linear velocity as:

(43) $$\overset{⌢}{v}=v_{\text{ec}}+\omega_{0}\times {\overset{⌢}{L}}_{1e}+M_{m}\omega_{0}\times b_{0}$$

Equation (42) can be further derived as:

(44) $$\frac{\partial f\left(\begin{array}{cc} {\hat{\gamma }}_{\text{tem}}, & {\hat{b}}_{0\text{tem}} \end{array}\right)}{\partial \gamma }=\overset{⌢}{v}\left({\hat{b}}_{0\text{tem}}\right)$$

Substituting Equation (27) into Equation (38), $\frac{\partial f\left(\begin{array}{cc} {\hat{\gamma }}_{\text{tem}}, & {\hat{b}}_{0\text{tem}} \end{array}\right)}{\partial b_{0}}$ can be determined as:

(45) $$\frac{\partial f\left(\begin{array}{cc} {\hat{\gamma }}_{\text{tem}}, & {\hat{b}}_{0\text{tem}} \end{array}\right)}{\partial b_{0}}=\left[1+M_{m}{\hat{\gamma }}_{\text{tem}}\right]{\tilde{\omega }}_{0}$$

Defining a robot angular velocity $\overset{⌢}{\omega }$ as:

(46) $$\overset{⌢}{\omega }=\left[1+M_{m}\gamma \right]\omega_{0}$$

Equation (45) can be further expressed as:

(47) $$\frac{\partial f\left(\begin{array}{cc} {\hat{\gamma }}_{\text{tem}}, & {\hat{b}}_{0\text{tem}} \end{array}\right)}{\partial b_{0}}=\tilde{\overset{⌢}{\omega }}\left({\hat{\gamma }}_{\text{tem}}\right)$$

Substituting Equations (44) and (47) into Equation (40), the estimation error can be calculated as:

(48) $$e=f\left(\begin{array}{cc} {\hat{\gamma }}_{\text{tem}}, & {\hat{b}}_{0\text{tem}} \end{array}\right)+\overset{⌢}{v}\left({\hat{b}}_{0\text{tem}}\right)\Delta \hat{\gamma }+\tilde{\overset{⌢}{\omega }}\left({\hat{\gamma }}_{\text{tem}}\right)\Delta {\hat{b}}_{0}-v_{e}$$

Defining the end-effector velocity calculated by temporarily identified mass properties as ${\hat{v}}_{\text{tem}}\left(\begin{array}{cc} {\hat{\gamma }}_{\text{tem}}, & {\hat{b}}_{0\text{tem}} \end{array}\right)$, Equation (48) can be further expressed as:

(49) $$e={\hat{v}}_{\text{tem}}\left(\begin{array}{cc} {\hat{\gamma }}_{\text{tem}}, & {\hat{b}}_{0\text{tem}} \end{array}\right)-v_{e}+{\overset{⌢}{v}}_{\text{ec}}\left({\hat{b}}_{0\text{tem}}\right)\Delta \hat{\gamma }+{\tilde{\overset{⌢}{\omega }}}_{0}\left({\hat{\gamma }}_{\text{tem}}\right)\Delta {\hat{b}}_{0}$$

According to Equation (41), once parameter errors $\Delta \hat{\gamma }$ and $\Delta {\hat{b}}_{0}$ are determined, the estimated mass properties $\hat{\gamma }$ and ${\hat{b}}_{0}$ can be simply identified. Since the number of the parameters to be identified is larger than the number of equations described by Equation (49), $\Delta \hat{\gamma }$ and $\Delta {\hat{b}}_{0}$ can’t be determined by simply assuming that the estimation error vector is ***0***. To solve this problem, other constraints need to be introduced.

Employing the least-squares technique, the total estimation error during the time interval $\left(\begin{array}{cc} t-\Delta t, & t \end{array}\right)$ is defined as:

(50) $$Q=\int_{t-\Delta t}^{t}e{\left(r\right)}^{T}e\left(r\right)dr$$

where Δt is the length of the integral interval. To ensure the total estimation error is minimized, first order partial differential equations of Q can be determined by:

(51) $$\{\begin{array}{l} \frac{\partial Q}{\partial \Delta \gamma }=2\int_{t-\Delta t}^{t}e{\left(r\right)}^{T}\frac{\partial e\left(r\right)}{\partial \Delta \gamma }dr=0\\ \frac{\partial Q}{\partial \Delta b_{0}}=2\int_{t-\Delta t}^{t}\frac{\partial e\left(r\right)}{\partial \Delta b_{0}}e\left(r\right)dr=0_{3\times 1} \end{array}$$

Substituting Equations (44), (47) and (48) into Equation (51), the following equations hold as:

(52) $$\{\begin{array}{l} \int_{t-\Delta t}^{t}{\overset{⌢}{v}}^{T}\overset{⌢}{v}dr\Delta \hat{\gamma }+\int_{t-\Delta t}^{t}{\overset{⌢}{v}}^{T}\tilde{\overset{⌢}{\omega }}dr\Delta {\hat{b}}_{0}=\int_{t-\Delta t}^{t}{\overset{⌢}{v}}^{T}\left(v_{e}-{\hat{v}}_{\text{etem}}\right)dr\\ \int_{t-\Delta t}^{t}\tilde{\overset{⌢}{\omega }}\overset{⌢}{v}dr\Delta \hat{\gamma }+\int_{t-\Delta t}^{t}\tilde{\overset{⌢}{\omega }}\tilde{\overset{⌢}{\omega }}dr\Delta {\hat{b}}_{0}=\int_{t-\Delta t}^{t}\tilde{\overset{⌢}{\omega }}\left(v_{e}-{\hat{v}}_{\text{etem}}\right)dr \end{array}$$

Defining the parameter error vector as:

(53) $$X=\left[\begin{array}{c} \Delta \hat{\gamma }\\ \Delta {\hat{b}}_{0} \end{array}\right]$$

according to Equation (52), the identification equation can be formed as:

(54) AX=B

where:

(55) $$A=\left[\begin{array}{cc} \int_{t-\Delta t}^{t}{\overset{⌢}{v}}^{T}\overset{⌢}{v}dr & \int_{t-\Delta t}^{t}{\overset{⌢}{v}}^{T}\tilde{\overset{⌢}{\omega }}dr\\ \int_{t-\Delta t}^{t}\tilde{\overset{⌢}{\omega }}\overset{⌢}{v}dr & \int_{t-\Delta t}^{t}\tilde{\overset{⌢}{\omega }}\tilde{\overset{⌢}{\omega }}dr \end{array}\right]$$

(56) $$B=\left[\begin{array}{c} \int_{t-\Delta t}^{t}{\overset{⌢}{v}}^{T}\left(v_{e}-v_{\text{enom}}\right)dr\\ \int_{t-\Delta t}^{t}\tilde{\overset{⌢}{\omega }}\left(v_{e}-v_{\text{enom}}\right)dr \end{array}\right]$$

According to Equation (54), the parameter error vector can be determined by:

(57) $$X=A^{-1}B$$

After all, the estimated mass properties $\hat{\gamma }$ and ${\hat{b}}_{0}$ can be computed according to Equations (41), (53) and (57).

Note that, the estimated mass properties are initialized by the nominal mass and centroid position in this work. After that, the temporarily identified mass properties must be updated in real time for more accurate estimations. In addition, the length of the integral interval has to be carefully designed according to the specified system properties and the practical mission requirements because the condition number of matrix A can be very large, even singular when Δt is too small. On the other hand, Δt cannot be too large either. Although the velocities as $\overset{⌢}{v}$ and $\overset{⌢}{\omega }$ are quite simple in calculation, longer integral intervals still require greater computations which are against the on-line application. Because the tuning process is based on the feedback of eye-in-hand camera images, which are usually quite noisy, the identification error is investigated to indicate the impacts of the measurement errors. The upper bound estimation of the identification error is given in the Appendix A of this paper.

## 4. Ground Testing Based on Hardware-in-the-Loop Simulation System

**Figure 4:**
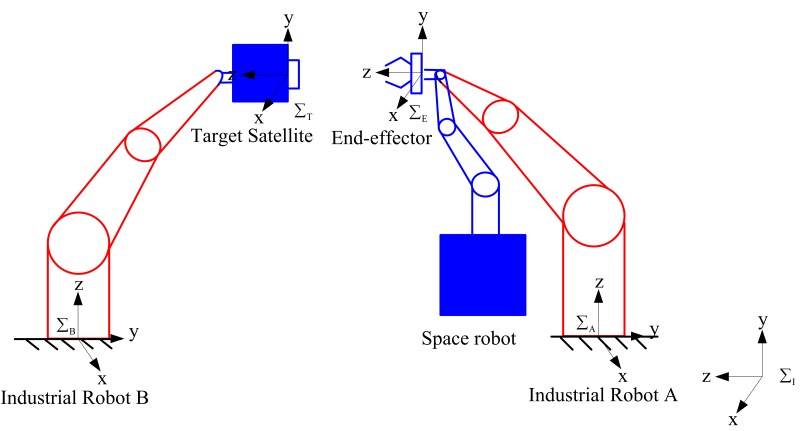
Kinematic equivalence diagram of hard-ware-in-the-loop simulation system.

Generally, space robot target capture methods are tested with ground test systems [34,35,36]. Thus a hardware-in-the-loop simulation system, illustrated by Figure 4, is employed for simulating the satellite capture process. Two industrial robots are used to simulate the space robot motions and the target satellite respectively: industrial robot A represents the space robot motions and the target satellite is mounted on industrial robot B.

To mimic the weightless condition in space, which are quite difficult to achieve in a ground environment, a space robot dynamics simulation program is established for calculating the space robot motions under microgravity. Since a carrier satellite was not available, gyro data is also output by this program. Moreover, a joint electronic simulator employing the same electronic interface with real space robot joints is used to simulate the robotic joint dynamics since the actual space robot is missing too. The eye-in-hand camera system and the space robot central controller are both real.

**Figure 5:**
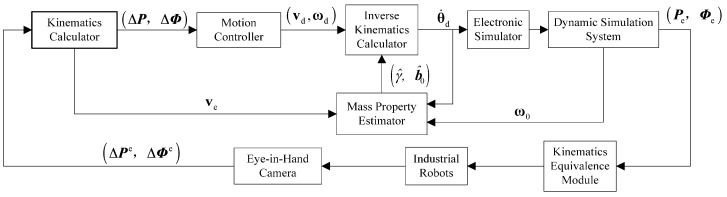
Block diagram of space robot ground test system.

The space robot hardware-in-the-loop simulation system consists of two industrial robots, the space robot motion controller, the inverse kinematics calculator, an electronic simulator, the space robot dynamics simulation system, the kinematic equivalence module, the eye-in-hand camera system and the mass property estimator. The space robot motion controller, inverse kinematics calculations and the proposed mass property estimation algorithm are all realized by the space robot central controller. The block diagram of designed experimental system is shown in Figure 5.

In the satellite capturing experimental system, the needed linear and angular velocities are calculated through the relative position and attitude measured by the vision system. Meanwhile, the mass property estimator determines the unknown parameters from the past joint trajectories and sensor information in real time. Then, the joint motions are planned based on the estimations and the desired end-effector motion. According to the joint motions, the electronic simulator determines the output torques of the joints. Then the motions of the space robot are simulated by the dynamic simulation system. Finally, the relative motions between the space robot and the target satellite are demonstrated by the industrial robots based on kinematic equivalence.

**Figure 6:**
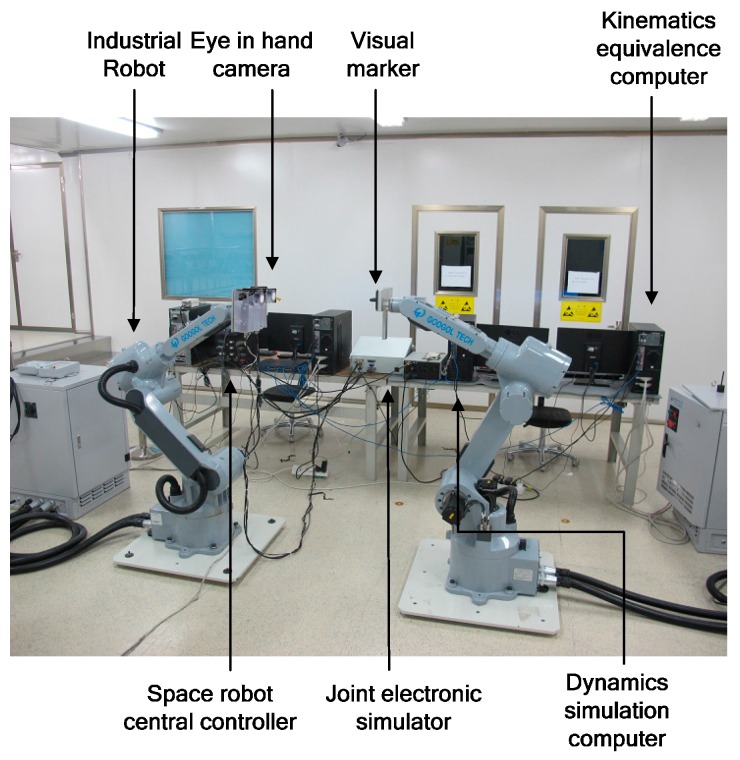
Laboratory with the space robot ground experimental system.

The instruction cycle of the designed space robot central controller is 250 ms. The joint electronic simulator’s control cycle is 25 ms. The required measurement accuracies of the eye-in-hand camera system are 1 mm in distance and 1 deg in orientation. The measurement frequency is 4 Hz. A picture of the laboratory setup is shown in Figure 6. Space robot kinematic and dynamic parameters are expressed as Table 1.

In this test, the space robot is required to move the end-effector from the initial position to the satellite with suitable posture. The nominal mass properties of carrier satellite are defined as:

(58) $$\{\begin{array}{l} m_{0nom}=680\left(\text{kg}\right)\\ b_{0nom}={\left[\begin{array}{ccc} 500 & 0 & 800 \end{array}\right]}^{T}\left(\text{mm}\right) \end{array}$$

Although the satellite mass is not necessary in the self-tuning control scheme, it is computed by $\hat{\gamma }$ for verifying the estimator. According to Equations (4) and (7), the estimated satellite mass can be calculated as:

(59) $${\hat{m}}_{0}=-\frac{1}{\hat{\gamma }}-M_{m}$$

Accordingly, the property estimation errors are computed as follows:

(60) $$\{\begin{array}{l} \delta m_{0}={\hat{m}}_{0}-m_{0}\\ \delta b_{0}={\hat{b}}_{0}-b_{0} \end{array}$$

**Figure 7:**
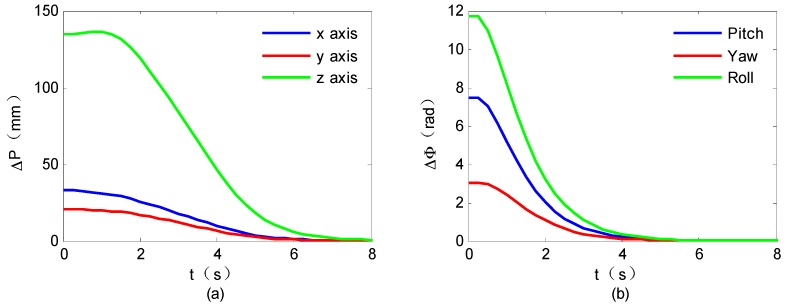
Closed-loop responses of space robot system: (**a**) Position errors; (**b**) Angle errors.

**Figure 8:**
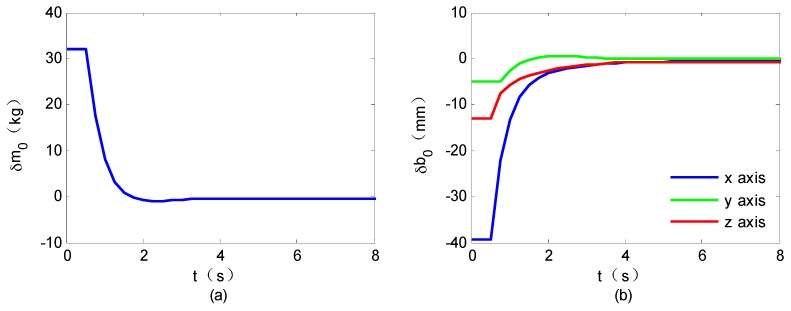
Property estimation errors: (**a**) estimation error of $m_{0}$; (**b**) estimation error of $b_{0}$.

To validate the proposed control scheme, both the closed-loop responses and the parameter estimation errors are tested by the ground experimental system. The experimental results are shown in Figure 7 and Figure 8.

The closed-loop responses are shown in Figure 7. It is seen that, the space robot end-effector can approach the target satellite smoothly with the desired attitude, illustrating the effectiveness of the proposed target capture control method. The mass property estimation errors are shown in Figure 8. They suggest that although the nominal mass properties are initialized with obvious errors, the estimated mass properties gradually approach the real values.

As the first attempt for estimating the satellite mass properties by eye-in-hand camera signals, the following conclusions can be made by comparing the proposed method with other existing identification methods:

(1) Compared with the propulsion-based methods as presented in [37], because the thrusters are not applied in the proposed method, no satellite fuel will be consumed.
(2) Unlike the direct torque-sensing method proposed in [38], this proposed method doesn’t demand any torque or acceleration measurements, not only in theory but also in engineering.
(3) Compared with the method based on measuring the reaction wheel motion rates presented in [32], which has difficulty estimating the satellite mass, the proposed method identifies both satellite mass and centroid position by adopting eye-in-hand camera signals and gyro information.
(4) Compared with the method presented in [33], which estimates the satellite mass properties base on sensing the satellite rotation and translation, this proposed method doesn’t require the linear velocity of the carrier satellite, which is usually integrated from accelerometer data and brings drifting errors.

## 5. Conclusions

Target satellite capture is a challenging problem, especially for space robots with unknown mass properties. Since most existing works for space robot motion control require accurate property values, new efforts are being made for handling such control problems including unknown parameters. In this paper, gyro and camera signals are adopted to improve the control performance. For this improved system, a novel space robot modelling technique is proposed. By this newly established model, the free-floating space robot is equivalent to a ground-fixed manipulator system, thus simplifying the issue. Accordingly, a self-tuning target capturing controller is designed taking unknown parameters into count. The control parameters are determined in real time by the estimator established based on the least-squares technique. The experimental results suggest that the designed space robot target capturing controller is effective. Because the proposed method does not demand accurate satellite mass properties, it can be applied when the fuel consumption is unknown or carrying an undetermined payload, etc. As further research, the proposed method has a potential to be applied in identifying other space robot parameters, such as manipulator link lengths, to cope with contingent requirements.

## Figures and Tables

**Table 1:** Kinematic and dynamic parameters of space robot.

Parameter (unit)	Base	Pole 1	Pole 2	Pole 3	Pole 4	Pole 5	Pole 6
M (kg)	648	1.5	9.6	1.5	9.0	1.5	10.5
$a_{x}$ (mm)	0	0	−493.5	0	289	0	−112
$a_{y}$ (mm)	0	0	56	−123	−123	127	0
$a_{z}$ (mm)	0	120	0	0	0	0	0
$b_{x}$ (mm)	539	0	−493.5	123	333	−123	−123
$b_{y}$ (mm)	5	189	−56	0	12	0	0
$b_{z}$ (mm)	813	0	0	0	0	0	0
$I_{\text{xx}}$ (kg∙m^2^)	198	$3.12\times 10^{-3}$	$3.71\times 10^{-2}$	$3.47\times 10^{-3}$	0.82	$3.42\times 10^{-3}$	0.91
$I_{\text{yy}}$ (kg∙m^2^)	198	$3.12\times 10^{-3}$	1.92	$3.47\times 10^{-3}$	0.64	$3.42\times 10^{-3}$	0.91
$I_{\text{zz}}$ (kg∙m^2^)	198	$3.12\times 10^{-3}$	1.92	$3.47\times 10^{-3}$	0.67	$3.42\times 10^{-3}$	0.11

## Appendix A

The measured velocity data is expressed as:

(A1) $$\{\begin{array}{l} {\bar{v}}_{e}=v_{e}+\Delta v_{e}\\ {\bar{\omega }}_{0}=\omega_{0}+\Delta \omega_{0} \end{array}$$

where $\Delta v_{e}$, as well as $\Delta \omega_{0}$, is the measurement error. According to Equations (43) and (46), the defined velocities are computed as:

(A2) $$\{\begin{array}{c} \bar{\overset{⌢}{v}}=v_{\text{ec}}-{\overset{⌢}{L}}_{0e}\left({\hat{b}}_{0\text{tem}}\right)\times {\bar{\omega }}_{0}\\ \bar{\overset{⌢}{\omega }}=\left[1+M_{m}{\hat{\gamma }}_{\text{tem}}\right]{\bar{\omega }}_{0} \end{array}$$

According to Equation (54), the identification equation is determined as:

(A3) $$\bar{A}\bar{X}=\bar{B}$$

where:

(A4) $$\bar{A}=A+\Delta A=\left[\begin{array}{cc} \int_{t-\Delta t}^{t}\bar{\overset{⌢}{v}}\cdot \bar{\overset{⌢}{v}}dr & -{\int_{t-\Delta t}^{t}\left(\bar{\overset{⌢}{\omega }}\times \bar{\overset{⌢}{v}}\right)}^{T}dr\\ \int_{t-\Delta t}^{t}\bar{\overset{⌢}{\omega }}\times \bar{\overset{⌢}{v}}dr & \int_{t-\Delta t}^{t}\bar{\overset{⌢}{\omega }}\times \left(\bar{\overset{⌢}{\omega }}\times \right)dr \end{array}\right]$$

(A5) $$\bar{B}=B+\Delta B=\left[\begin{array}{c} \int_{t-\Delta t}^{t}\bar{\overset{⌢}{v}}\cdot \left({\bar{v}}_{e}-v_{\text{enom}}\right)dr\\ \int_{t-\Delta t}^{t}\bar{\overset{⌢}{\omega }}\times \left({\bar{v}}_{e}-v_{\text{enom}}\right)dr \end{array}\right]$$

and $\bar{X}$ is the identification result with the error δX; ΔA and ΔB are the errors of A and B caused by the measurement errors $\Delta v_{e}$ and $\Delta \omega_{0}$. The error matrixes ΔA and ΔB is expressed as follows:

(A6) $$\{\begin{array}{l} \Delta A=\varepsilon_{A}E_{A}\\ \Delta B=\varepsilon_{B}E_{B} \end{array}$$

where:

(A7) $$\{\begin{array}{l} \varepsilon_{A}=\left\|\Delta A\right\|\\ \varepsilon_{B}=\left\|\Delta B\right\|\\ \left\|E_{A}\right\|=\left\|E_{B}\right\|=1 \end{array}$$

In accordance with the theory presented in [33], the upper bound of the error for the identification problem represented by Equation (A3) can be estimated by:

(A8) $$\left\|\delta X\right\|\leq {\left\|A^{-1}\right\|}^{2}\left\|r\right\|\varepsilon_{A}+\left\|A^{-1}\right\|\left(\left\|X\right\|\varepsilon_{A}+\varepsilon_{B}\right)+N_{1\varepsilon }$$

and the upper bound of the relative error in the identified parameters is:

(A9) $$\frac{\left\|\delta X\right\|}{\left\|X\right\|}\leq {\left(\kappa \left(A\right)\right)}^{2}\frac{\varepsilon_{A}}{\left\|A\right\|}\zeta +\kappa \left(A\right)\left(\frac{\varepsilon_{A}}{\left\|A\right\|}+\frac{\varepsilon_{B}}{\left\|B\right\|}\gamma \right)+N_{2\varepsilon }$$

where r is the residual vector; κ is the condition number; $N_{1\varepsilon }$, as well as $N_{2\varepsilon }$, represents the second- and higher-order terms of ε, which can be practically ignored. They and others are computed as follows:

(A10) $$\left\{ \begin{array}{l} r=B-AX\\ \kappa \left(A\right)=\left\|A^{-1}\right\|\left\|A\right\|\\ \zeta =\frac{\left\|r\right\|}{\left\|A\right\|\left\|X\right\|}\\ \gamma =\frac{\left\|B\right\|}{\left\|A\right\|\left\|X\right\|} \end{array}\right.$$

To determine the error bounds, ΔA as well as ΔB should be computed in terms of the measurement errors $\Delta v_{e}$ and $\Delta \omega_{0}$. Substituting Equations (A1) and (A2) into Equations (A4) and (A5), ΔA and ΔB are expressed as:

(A11) $$\Delta A=\left[\begin{array}{cc} \int_{t-\Delta t}^{t}\Delta \overset{⌢}{v}\cdot \left(2\bar{\overset{⌢}{v}}-\Delta \overset{⌢}{v}\right)dr & -{\int_{t-\Delta t}^{t}\left(\Delta \overset{⌢}{\omega }\times \bar{\overset{⌢}{v}}+\bar{\overset{⌢}{\omega }}\times \Delta \overset{⌢}{v}-\Delta \overset{⌢}{\omega }\times \Delta \overset{⌢}{v}\right)}^{T}dr\\ \int_{t-\Delta t}^{t}\left(\Delta \overset{⌢}{\omega }\times \bar{\overset{⌢}{v}}+\bar{\overset{⌢}{\omega }}\times \Delta \overset{⌢}{v}-\Delta \overset{⌢}{\omega }\times \Delta \overset{⌢}{v}\right)dr & \int_{t-\Delta t}^{t}\left(\bar{\overset{⌢}{\omega }}\times \Delta \overset{⌢}{\omega }\times +\Delta \overset{⌢}{\omega }\times \bar{\overset{⌢}{\omega }}\times -\Delta \overset{⌢}{\omega }\times \Delta \overset{⌢}{\omega }\times \right)dr \end{array}\right]$$

(A12) $$\Delta B=\left[\begin{array}{c} \int_{t-\Delta t}^{t}\left(\bar{\overset{⌢}{v}}\cdot \Delta v_{e}+\Delta \overset{⌢}{v}\cdot \delta v_{e}-\Delta \overset{⌢}{v}\cdot \Delta v_{e}\right)dr\\ \int_{t-\Delta t}^{t}\left(\bar{\overset{⌢}{\omega }}\times \Delta v_{e}+\Delta \overset{⌢}{\omega }\times \delta v_{e}-\Delta \overset{⌢}{\omega }\times \Delta v_{e}\right)dr \end{array}\right]$$

where:

(A13) $$\left\{ \begin{array}{l} \Delta \overset{⌢}{v}=\bar{\overset{⌢}{v}}-\overset{⌢}{v}=-{\overset{⌢}{L}}_{0e}\times \Delta \omega_{0}\\ \Delta \overset{⌢}{\omega }=\bar{\overset{⌢}{\omega }}-\overset{⌢}{\omega }=\left[1+M_{m}{\hat{\gamma }}_{\text{tem}}\right]\Delta \omega_{0}\\ \delta v_{e}={\bar{v}}_{e}-v_{\text{enom}} \end{array}\right.$$

Ignoring the second-order terms, ΔA and ΔB are rewritten as functions of $\Delta \omega_{0}$ and $\Delta v_{e}$:

(A14) $$\Delta A=\left[\begin{array}{cc} -\int_{t-\Delta t}^{t}\left(2{\bar{\overset{⌢}{v}}}^{T}{\tilde{\overset{⌢}{L}}}_{0e}\right)\Delta \omega_{0}dr & \int_{t-\Delta t}^{t}{\left\{\left[\left(1+M_{m}{\hat{\gamma }}_{\text{tem}}\right)\tilde{\bar{\overset{⌢}{v}}}+\tilde{\bar{\overset{⌢}{\omega }}}{\tilde{\overset{⌢}{L}}}_{0e}\right]\Delta \omega_{0}\right\}}^{T}dr\\ -\int_{t-\Delta t}^{t}\left[\left(1+M_{m}{\hat{\gamma }}_{\text{tem}}\right)\tilde{\bar{\overset{⌢}{v}}}+\tilde{\bar{\overset{⌢}{\omega }}}{\tilde{\overset{⌢}{L}}}_{0e}\right]\Delta \omega_{0}dr & \left(1+M_{m}{\hat{\gamma }}_{\text{tem}}\right)\int_{t-\Delta t}^{t}\left[\tilde{\bar{\overset{⌢}{\omega }}}\Delta {\tilde{\omega }}_{0}+{\left(\tilde{\bar{\overset{⌢}{\omega }}}\Delta {\tilde{\omega }}_{0}\right)}^{T}\right]dr \end{array}\right]$$

(A15) $$\Delta B=\left[\begin{array}{c} \int_{t-\Delta t}^{t}{\bar{\overset{⌢}{v}}}^{T}\Delta v_{e}-\delta v_{e}^{T}{\tilde{\overset{⌢}{L}}}_{0e}\Delta \omega_{0}dr\\ \int_{t-\Delta t}^{t}\tilde{\bar{\overset{⌢}{\omega }}}\Delta v_{e}-\left[1+M_{m}{\hat{\gamma }}_{\text{tem}}\right]\delta {\tilde{v}}_{e}\Delta \omega_{0}dr \end{array}\right]$$
